# The MacArthur-Bates Communicative Development Inventories: updates from the CDI Advisory Board

**DOI:** 10.3389/fpsyg.2023.1170303

**Published:** 2023-05-31

**Authors:** Virginia A. Marchman, Philip S. Dale

**Affiliations:** ^1^Department of Psychology, Stanford University, Stanford, CA, United States; ^2^Department of Speech and Hearing Sciences, University of New Mexico, Albuquerque, NM, United States

**Keywords:** MacArthur-Bates CDI, parent report, language development, Wordbank, Web-CDI, CDI-CAT

## 1. Introduction

Beginning with the classic diary studies (e.g., Stern and Stern, 1907; Leopold, 1949), parents (and other caregivers)^1^ have been a source of valuable insights on their child's early language and communicative skills. The logic behind parent report is simple. Parents are generally keenly aware of their child's behaviors and their impressions are based on hours of observation in diverse settings, rather than the brief time available in a standard clinic or office visit. Moreover, their reports are less likely to be influenced by factors that may mask a child's “true” abilities in the laboratory or clinic (e.g., child non-compliance).

The MacArthur-Bates Communicative Development Inventories (CDIs) evolved from early efforts to harness this parental expertise in the 1970 and 1980's by Elizabeth Bates and colleagues (Bates et al., 1975, 1988). These instruments were further developed in the mid-1990's and beyond (Fenson et al., 1993, 1994, 2007), first for families with children learning American English, followed shortly by Mexican Spanish (Jackson-Maldonado et al., 1993, 2003) and Italian (Caselli and Casadio, 1995). For Bates, the main keys to success for parent report were to ask parents to choose from a list of example words or behaviors (e.g., recognition), rather than to recall them from memory, and to focus on emerging, salient behaviors, rather than to require retrospective reports.

Most versions of the CDIs come in two levels/forms. The Words and Gestures (CDI:WG) form, for children 8–18 months, asks parents to indicate on a vocabulary checklist which words or phrases their child “understands” or “understands and says,” and to choose among examples of early communicative and symbolic gestures. The Words and Sentences (CDI:WS) form, for children 16–30 months, asks parents to select the words their child produces on their own, and also to indicate their child's use of grammatical forms (e.g., plural -s) and word combinations (e.g., “mommy sock”). While these original “long form” versions provide a comprehensive picture of early language, they typically require 20–30 min or longer for the parent to complete. Consequently, short form versions focusing only on vocabulary have been developed for when a comprehensive assessment is not needed or parental time commitment is limited (Fenson et al., 2000; Jackson-Maldonado et al., 2013). Versions appropriate for slightly older children, the CDI-III, have also been developed (e.g., Dale et al., 2023; Jackson-Maldonado et al., 2023).

The CDIs have been used to explore many questions relevant to researchers and clinicians, for example, to what extent do demographic or environmental factors influence language development? Does a low score on the CDI predict continued or future language delays? Most analyses rely on aggregate scores from the vocabulary checklist, e.g., total words understood or total words produced. But individual item responses can also be analyzed, investigating questions such as the relative difficulty of words, or whether some words are more likely to be learned by boys vs. girls (Braginsky et al., 2019).

In the late 1980's, the developers of the American English and Mexican Spanish CDIs came together to form an Advisory Board. For more than 25 years, the Board has used proceeds from the sales of these instruments, distributed by Brookes Publishing Co (https://brookespublishing.com/product/cdi/), to support a variety of initiatives in the U.S. and internationally. Thanks to the strong interest and considerable effort of researchers around the globe, the Board has authorized versions of CDIs in more than 100 languages, with each instrument adapted (not just translated) to fit the linguistic and sociocultural features of that language. These important contributions are too numerous to mention individually, so we invite readers to peruse the full list here: https://mb-cdi.stanford.edu/adaptations.html. It is gratifying to reflect on the role of the CDIs in the crosslinguistic child language landscape, making contributions to our understanding of the normative course, as well as the individual differences, that characterize early language development (e.g., Bornstein et al., 2004; Bleses et al., 2008; Tardif et al., 2008; Jørgensen et al., 2010; Frank et al., 2021). At the same time, we acknowledge that significant gaps remain in the availability of CDIs across the world's languages (Kidd and Garcia, 2022).

In this Opinion, we seek to remember Liz Bates and the contributions that she made by briefly reviewing four recent significant innovations directed by the MacArthur-Bates CDI Advisory Board. First, we overview Wordbank, an open repository of CDI administrations from dozens of languages (Frank et al., 2017, 2021). Second, we report on an online platform for administration and scoring called Web-CDI (deMayo et al., 2021). Third, we discuss the development of a new, computer-adaptive testing instrument, the CDI-CAT (Kachergis et al., 2022). Finally, we announce the expanded and improved normative data for the American English long forms included in the 3rd Edition of the User's Guide and Technical Manual (Marchman et al., 2023).

## 2. Four major initiatives

### 2.1. Harnessing the power of “big data”

Many samples of CDI data to date have been limited in both size and scope because, with few exceptions, they were collected at a single site or laboratory. When data are combined across laboratories, the resulting datasets are not only larger, but are also likely to be considerably more representative along key dimensions (e.g., socioeconomic status). Building the infrastructure to enable data sharing across laboratories is non-trivial, but an enterprise that has a history in our field, for example, ChiLDES and CLEX (MacWhinney, 2000; Jørgensen et al., 2010). Inspired by this work, Michael Frank and his team developed Wordbank (http://wordbank.stanford.edu, Frank et al., 2017), a structured database of cross-linguistic CDI data currently consisting of more than 80,000 CDI administrations in 38 different languages. Such amazing progress would not have been possible without the many researchers who contributed their data (http://wordbank.stanford.edu/contributors). Wordbank also provides a powerful, browseable web interface that allows interactive exploration at the level of individual children (aggregating across words) and of individual words (aggregating across children). Recent analyses reveal remarkable insights into both the consistency and variability in early language development across languages (Braginsky et al., 2019; Frank et al., 2021). In just a few short years, Wordbank has become an invaluable tool with many research and teaching applications.

### 2.2. Moving beyond paper-and-pencil

Traditionally, CDIs are completed on paper: parents check off responses using pencil/pen and scores are later hand-tabulated. Today many prefer to engage with an electronic or online format on a laptop, tablet, or smart phone. Electronic administration eliminates postage costs, does not require face-to-face contact, and minimizes the chance of lost forms. Moreover, scoring is simplified since responses need not be transferred from the paper into an electronic format. Two options for electronic administration are available through Brookes Publishing Co. First, users can purchase fillable pdfs of the American English and Mexican Spanish forms, which can be emailed to families. Tabulation of scores is straightforward, but requires additional tools, such as Excel. A second option is Web-CDI, which offers a comprehensive online administration, data management, and scoring platform. Similar to platforms in other languages (Kristoffersen et al., 2013; Gendler-Shalev and Dromi, 2022), users share URLs (web links to a researcher's or clinician's own Web-CDI study) via email or social media, facilitating access to families at a distance. Importantly, Web-CDI's infrastructure ensures anonymity and participant privacy. Moreover, pictorial instructions are provided to facilitate uptake of critical information (see http://mb-cdi.stanford.edu/about). End-users can download tabulated summary scores, percentiles, and individual item responses automatically, facilitating analyses at both the child and item levels.

A recent analysis showed that demographic trends are similar for the American English long forms collected with Web-CDI and paper (deMayo et al., 2021). Moreover, Web-CDI has been successfully used to recruit American English-speaking families from diverse backgrounds, offering hope that the platform may help increase representation across ethnic/racial and educational groups. Managed in parallel at Stanford University and the Max Planck Institute, CDIs for American English, Mexican Spanish, Canadian French, Korean, Hebrew, Dutch, and Argentinian Spanish are currently available in Web-CDI, and there is a straightforward procedure for adding more languages.

### 2.3. Introducing the CDI-CAT

The vocabulary checklists of the CDI forms typically include hundreds of words, yielding a comprehensive view of children's vocabulary across many different lexical categories. However, asking about many words that a child is unlikely to know is inefficient and provides little information about the specific child relative to their peers. Computerized adaptive testing (CAT; van der Linden and Glas, 2009) offers an alternative approach. Each parent responds to an individualized list of words, each one selected based on their responses to the previous items. Scoring is computed using a statistical model based on Item Response Theory (IRT). Kachergis et al. (2022) reports on the development and testing of CDI-CATs for both comprehension and production vocabulary in both American English and Mexican Spanish (see also Kachergis and Dale, 2023). Like CATs in other languages (Mayor and Mani, 2019; Mieszkowska et al., 2022), even very short American English CDI-CATs (20–25 items) recovered participant abilities very well with little bias across ages. Moreover, a validation study with more than 200 children whose parents completed both the American English CDI-CAT and the American English CDI:WS showed a very strong correlation (*r* = 0.92). CDI-CATs for vocabulary production in American English and Mexican Spanish are available the spring of 2023 within Web-CDI. CDI-CATs are currently being developed in other languages (e.g., French) with others forthcoming.

### 2.4. New and improved normative information for the American English forms

**Figure 1:**
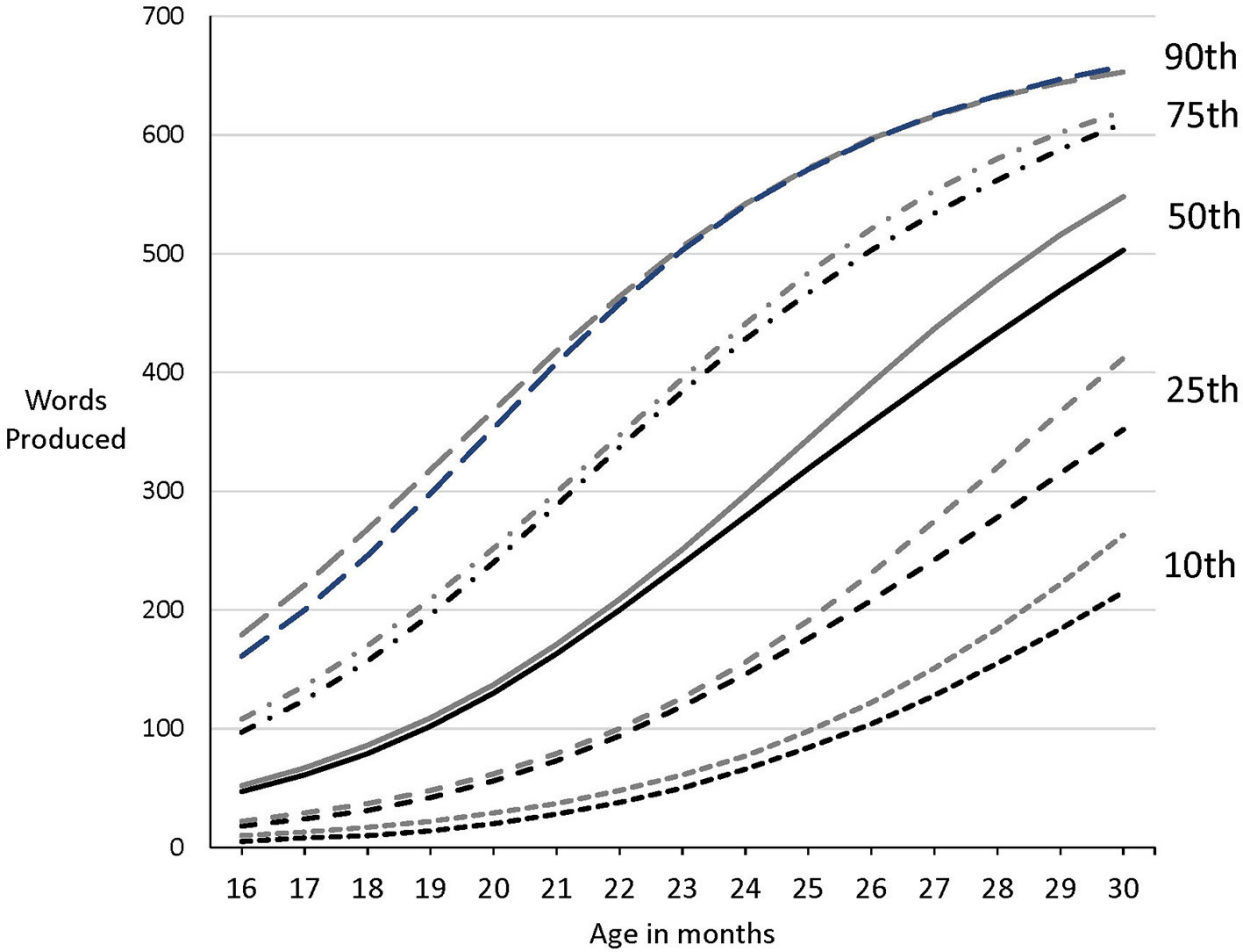
Percentile values (10, 25, 50, 75, and 90th) for words produced on the American English MacArthur-Bates Communicative Development Inventory: Words and Sentences form from Fenson et al. (2007; gray lines) and Marchman et al. (2023; dark lines).

Percentile scores for the major CDI measures place individual children in relation to a large norming sample. Unfortunately, the norming data for the American English long forms (Fenson et al., 2007) were not representative of the educational, racial, and ethnic distributions of the U.S. population. To remedy this situation, more than 4,000 additional CDI administrations have been added to the norming sample, yielding a sample of more than 6,500 children. Data were contributed by a consortium of researchers who used Web-CDI for their own independent research enterprises as well as via targeted online efforts, e.g., Facebook, to reach a broad, demographically diverse sample of caregivers. In addition, we statistically adjusted the data with raking, a technique for reweighting survey data (Lumley, 2020) in the R statistical package (R Core Team, 2020) to achieve a sample distribution that more closely resembled the demographic makeup of the target population. We used 2020 U.S. Census data^2^ for race, ethnicity, and caregiver (maternal) education to derive the weights. Models were fit using generalized additive models in the Beta distribution family (GAMLSS, Stasinopoulos et al., 2017), a technique that is more sophisticated than that used in earlier versions. These innovations are important because a norming sample that is biased toward more educated and otherwise advantaged families results in norms that are too high, and therefore, may over-classify late talkers. As illustrated in Figure 1, the new norms generally show lower scores, especially for children who fall < 90th percentile and who are older than 24 months of age. It is hoped that these types of statistical solutions will be informative to others who are interested in increasing the representativeness of their norming samples. These new normative data are available in Marchman et al. (2023).

## 3. What's next?

There is still much to be done. One important area is facilitating the administrative, analytic, and reporting practices that best serve children learning more than one language. Joining ongoing discussions (e.g., Gatt et al., 2015) and following from key research in this area (e.g., O'Toole et al., 2017), a new chapter on this topic is included in the new manual (Weisleder et al., 2023), which makes recommendations for best practices in assessment and reporting. We also look forward to efforts that stretch the limits of the parent report methodology to more effectively accommodate respondents with low-literacy or limited experience with electronic devices (e.g., Alcock et al., 2015). We also commend ongoing efforts to apply the parent report methodology to older children, as well as beyond the home context (e.g., Morford and Carlson, 2011; Eriksson, 2017; Bleses et al., 2018; Tulviste and Schults, 2020; Kas et al., 2022). The MacArthur-Bates CDI Advisory Board is committed to continuing to strengthen our knowledge and efficacy in these and other domains and welcomes the efforts of scholars around the world in further expanding the availability of CDIs worldwide.

## 4. Conclusion

In 2023, it will be 20 years since the untimely passing of Elizabeth Bates. In this Opinion, we have sought to honor Liz's memory by highlighting a few recent developments in parent report methodology spearheaded by the MacArthur-Bates CDI Advisory Board. We hope that readers of this special issue will appreciate hearing about our continuing efforts to build on her legacy by strengthening cross-laboratory and cross-linguistic collaboration, improving data administration and management techniques, and expanding the representativeness of normative data. We know that these initiatives represent only a few of the CDI-related activities that are ongoing in the child language community and acknowledge that there is still much more for all of us to do. We look forward to many more years of collaborations with the international community to improve and expand parent report as a useful tool for the fields of child language and developmental psycholinguistics.

## Author contributions

VM and PD contributed equally to the drafting of this manuscript. All authors contributed to manuscript revision, and have read and approved the submitted version.

## Footnotes

^1^We use the term “parent” to refer to any individual who takes care of the child on a regular basis, which could include parents, grandparents, step-parents, and others.

^2^https://www.census.gov, 2020.

## Editor's note

Maria-José Ezeizabarrena edited the article in collaboration with Melita Kovacevic, University of Zagreb, Zagreb, Croatia.
